# Worry about prostate cancer and risk perception among middle-aged men: results from the PROBASE trial

**DOI:** 10.1007/s10865-025-00559-w

**Published:** 2025-03-05

**Authors:** Valentin H. Meissner, Andreas Dinkel, Martina Kron, Stefan Schiele, Matthias Jahnen, Jale Lakes, Jan Philipp Radtke, Markus A. Kuczyk, Nina N. Harke, Jürgen Debus, Christoph A. Fink, Gerald Antoch, Lars Schimmöller, Glen Kristiansen, Agne Krilaviciute, Petra Seibold, Sabine Behrens, Axel Benner, Christian Arsov, Boris Hadaschik, Nikolaus Becker, Rudolf Kaaks, Peter Albers, Jürgen E. Gschwend, Kathleen Herkommer

**Affiliations:** 1 Department of Urology, Klinikum rechts der Isar, School of Medicine and Health, Technical University of Munich, Ismaninger Str. 22, 81675 Munich, Germany; 2 Department of Psychosomatic Medicine and Psychotherapy, Klinikum rechts der Isar, School of Medicine and Health, Technical University of Munich, Munich, Germany; 3 Institute of Epidemiology and Medical Biometrics, University of Ulm, Ulm, Germany; 4 Department of Urology, University Hospital, Medical Faculty, Heinrich-Heine University Duesseldorf, Duesseldorf, Germany; 5 Department of Urology, Hannover Medical School, Hannover, Germany; 6 Department of Radiation Oncology, Heidelberg University Hospital, Ruprecht Karls University Heidelberg, Heidelberg, Germany; 7 Department of Diagnostic and Interventional Radiology, Medical Faculty, Duesseldorf, Heinrich-Heine University Duesseldorf, Duesseldorf, Germany; 8 Institute of Pathology, University Hospital Bonn, Bonn, Germany; 9 Division of Personalized Early Detection of Prostate Cancer, German Cancer Research Center (DKFZ), Heidelberg, Germany; 10 Division of Cancer Epidemiology, German Cancer Research Center (DKFZ), Heidelberg, Germany; 11 Division of Biostatistics, German Cancer Research Center (DKFZ), Heidelberg, Germany; 12 Department of Urology, University of Duisburg-Essen and German Cancer Consortium (dktk), University Hospital Essen, Essen, Germany

**Keywords:** Absolute risk perception, Cancer worry, Comparative risk perception, Family history, Lower urinary tract symptoms, Prostate cancer

## Abstract

Cancer worry and risk perception are relevant psychological factors that influence preventive health behaviors. Therefore, a thorough understanding of the factors that impact their occurrence and manifestation is critical. The objective of this study was to assess prevalence and factors associated with worry about prostate cancer (PCa) and absolute/comparative risk perception in a community-based sample of 45-year-old men. Data were collected within the German PCa screening trial PROBASE. Variables were assessed by self-report questionnaires and a clinical interview. Worry about PCa and absolute/comparative risk perception were assessed each on a 5-point-Likert scale. Multivariable logistic regression was used to identify factors associated with the outcomes of interest. Data were available for 33,476 (72.0%) of 46,495 men at enrollment. 7.3% had sometimes/(very) often worry about PCa. 3.7% and 9.9% perceived their absolute risk and comparative risk as somewhat high/very high and somewhat higher/much higher, respectively. 18.8% reported a positive PCa family history. Important factors of worry about PCa, absolute risk perception, and comparative risk perception were lower urinary tract symptoms (LUTS) (OR 3.00, 95% CI 2.63–3.42; OR 2.09, CI 1.71–2.56; OR 2.41, CI 2.10–2.76) and a positive PCa family history (OR 2.35, CI 2.08–2.65; OR 15.13, CI 12.73–17.97; OR 9.69, CI 8.76–10.72). A positive history of urological (OR 3.85, CI 2.63–5.63) and non-urological cancers (OR 1.97, CI 1.52–2.54) were associated with a higher comparative risk perception. In conclusion, worry about PCa and risk perception are influenced by non-cancer-related symptoms as well as by a positive PCa family history. These findings need to be addressed in risk communication with patients.

**Supplementary Information:**

The online version contains supplementary material available at 10.1007/s10865-025-00559-w.

## Introduction

Prostate cancer (PCa) is the second most common cancer in men worldwide (Bray et al., 2018) and most often diagnosed following an elevated prostate-specific antigen (PSA) level (Cooperberg et al., 2005). However, there is uncertainty about the benefits of PCa screening due to an elevated risk of overdiagnosis (Brawley, 2012). Patients and their health care providers must weigh benefits, uncertainties, and risks of PCa screening resulting in a complex decision-making. Additionally, conflicting recommendations across various health authorities (Grossman et al., 2018; Mottet et al., 2017) increase this complexity and men with a family history of PCa must be aware of their elevated risk in their decision-making process. For instance, risk perceptions play an important role in many theories on health behavior including the Protection Motivation Theory (Rogers, 1975) and the Health Belief Model (Rosenstock et al., 1988) where higher perceived risk leads to more preventive health behavior.

Personal risk estimates vary significantly depending on whether individuals are rating their subjective risk alone (absolute risk perception) or relative to a standard (comparative risk perception), such as the risk of other individuals. Absolute and comparative risk perception of cancer appear to be relatively independent constructs, as suggested by their modest correlations. Moreover, absolute and comparative risk perceptions explained a different portion of the variance in cancer worry (Lipkus et al., 2000). For instance, comparative risk perception is related to affect, behavior, and health information processing even when controlling for absolute risk perceptions (Lipkus et al., 2005; Radcliffe & Klein, 2002). Among participants in a national survey reporting risk perceptions and cancer worry analyses showed that absolute and comparative risk perceptions were independent predictors of worry across all cancer sites (i.e., breast, colon, and prostate). However, absolute risk perceptions were more predictive than comparative risk perceptions of worry for women, but not for men (Zajac et al., 2006), emphasizing significant differences in these two constructs.

Similarly, other key factors in health behaviors and attitudes toward preventive health care are affective or experiential perceptions of vulnerability (e.g., cancer worry), even when controlling for cognitive perceptions (Jensen et al., 2010; Moser et al., 2007). The relationship between worry and behavior seems logical since affective appeals have been shown to motivate health behavior (Witte, 1998). Affective perceptions may either influence or be influenced by cognitive perceptions. If this is indeed the case, it has significant implications for the development of effective communication strategies aimed at promoting health-enhancing behaviors. For example, an association between perceived risk of PCa and worry about PCa has been described in a large sample of participants undergoing PCa screening (Cohen et al., 2003). Furthermore, in individuals with a positive family history of PCa higher perceived risk of PCa was associated with a higher likelihood of cancer-related worries affecting daily life (Bratt et al., 2000). While the evidence clearly indicates an association between risk perception of PCa and worry about the disease, the causal direction remains unclear. Some researchers conceptualized risk perception as antecedent of cancer worry (Schnur et al., 2006; Zajac et al., 2006), which has been shown in the context of other illnesses (DiLorenzo et al., 2006). However, the opposite causal direction has also been demonstrated by conceptual frameworks and empirical evidence, suggesting worry as a predictor of perceived risk for developing cancer (Hay et al., 2006; Slovic et al., 2004). The Tripartite Model of Risk Perception (TRIRISK) distinguishes among deliberative, experiential, and affective components of risk perception. In this framework cancer worry represents an aspect of risk perceptions, not a distinct model component (Ferrer et al., 2016). Nevertheless, both risk perception and cancer worry are well investigated as predictors of preventive health behavior such as PCa screening (Beebe-Dimmer et al., 2004; McDowell et al., 2009; Vadaparampil et al., 2004), and younger age, decreased subjective health, presence of physical symptoms, and a positive family history of cancer emerged most consistently as factors associated with higher cancer worry and risk perception (Hay et al., 2006; Hidalgo et al., 2015; Konings et al., 2017; Montgomery et al., 2003; Robb et al., 2004).

With regard to PCa, a British registry-based study examined worry about PCa in a sample of first-degree relatives of PCa patients. Higher health anxiety, higher subjective stress, and higher perceived risk reliably predicted higher worry about PCa (Sweetman et al., 2006), and the number of relatives deceased due to PCa is associated with worry about PCa among men in families with hereditary PCa (Bratt et al., 2000). With regard to risk perception, previous studies have found greater risk perceptions in men with a positive family history of PCa, whereas increasing age, which is a major risk factor for PCa, was associated with decreasing risk perceptions (McDowell et al., 2013; Schnur et al., 2006).

Taken together, despite the large number of studies investigating factors associated with preventive health behavior such as cancer worry and risk perceptions, available research concerning factors associated with cancer worry and risk perception, especially in PCa, is quite outdated and mainly limited to samples with a positive family history. Furthermore, recent research on this topic as well as studies investigating large community-based samples are lacking, while they are available for other cancers such as breast, colon and lung cancer (Hay et al., 2006; Lebrett et al., 2022; Zajac et al., 2006). Hence, the objective of this study was to investigate factors associated with worry about PCa and risk perception in a community-based sample of more than 30,000 45-year-old men who participated in a prospective risk-adapted PCa screening trial. Additionally, due to the large sample size and comprehensive data collection, it was possible to control for a broad set of sociodemographic, lifestyle, clinical, and psychological factors as well as family history.

## Methods

### Study procedure

Data for this study were collected within the ongoing German PCa screening trial PROBASE (Risk-adapted prostate cancer early detection study based on a „baseline“ PSA value in young men - a prospective multicenter randomized trial), which investigates the concept of risk-adapted PSA screening. 45-year-old men were recruited using random samples from the local population registers up to 100 km around the four study areas Dusseldorf, Hannover, Heidelberg, and Munich. The participants were randomized (1:1) into two study arms. Baseline PSA is measured in participants of study arm A at age 45, participants of study arm B receive their baseline PSA test at age 50. Written informed consent was obtained from all individuals prior to participation. The study was approved by the ethics committees of the four study centers. Further details on the PROBASE trial design have previously been described (Arsov et al., 2013, 2022).

46,495 men (study arm A and B) aged 45 years were recruited within the PROBASE trial between April 2014 and December 2019, irrespective of the aforementioned PSA measurement. Sociodemographic, clinical, lifestyle, and psychological factors as well as information about previous PSA testing/digital rectal examination, and family history were assessed by self-report questionnaires and a clinical interview. Inclusion criteria for this analysis were (I) being White and (II) having answered the questions on worry about PCa, absolute risk perception, and comparative risk perception at enrollment. The final reason to exclude men with ancestry other than White was not only due to a very low number of study participants, but also because response rates were significantly lower compared to White men. For instance, from 441 men with African ancestry only 131 responded to questions on worry about PCa, absolute risk perception, and comparative risk perception (mandatory inclusion criteria), which is a response rate of less than 30%. Compared to White men the response rate was significantly lower (33,476/45,025 = 74.3%). The same applied to men with Asian ancestry (181/447 = 40.5%). 33,476 men (72.0%) of the 46,495 men recruited within the PROBASE trial fulfilled the inclusion criteria. Baseline characteristics as well as prevalence rates of the target variables (i.e., worry about PCa, absolute risk perceptions, and comparative risk perception) were assessed in this population (Tables 1 and 2). Due to the large number of variables assessed, missing values for some variables were unavoidable. Taking missing data in further variables into account, 21,227 (45.7%) men were available for the final regression analyses. A detailed dropout analysis including numbers of missing variables is displayed in Supplementary Table 1. The dropout analysis revealed only marginal differences (all < 8%) between the two groups. The biggest differences were found in level of education and subjective economic situation. Here, excluded men had slightly more often a lower level of education (16.0% vs. 10.5%) and reported a poorer subjective economic situation (29.9% vs. 21.3%). Notably, all lifestyle, clinical, and especially psychological factors as well as family history showed no to only marginal differences (< 3.5%) between the two groups.

## Measures

### Sociodemographic factors

A self-report sheet was used to assess sociodemographic factors such as *level of education* (high (higher than high school) vs. intermediate (high school) vs. low (lower than high school)), living in a *partnership* (yes vs. no), type of *health insurance* (private vs. statutory), and *subjective economic situation* (good vs. poor).

### Lifestyle factors

Height, weight, and waist circumference were measured during the clinical interview and *Body Mass Index* (BMI) was calculated. Obesity was defined as a BMI ≥ 30 kg/m^2^ and central obesity was defined as a *waist circumference* > 102 cm, according to the World Health Organization guidelines (World Health Organization. (2011) Waist circumference and waist–hip ratio: report of a WHO expert consultation). Further lifestyle factors included *alcohol consumption* (high (≥ 3 alcoholic beverages on ≥ 5 days/week) vs. low to moderate/no), *active smoking* (yes (current smoker) vs. no (non-smoker/former smoker), and *physical activity* (yes (≥ 2 times/week for a minimum of 30 min including mild exercises like walking and gardening) vs. no). *Alcohol consumption* was assessed with questions from the Alcohol Use Disorders Identification Test (AUDIT) (Saunders et al., 1993), a widely used assessment tool in healthcare settings for the measurement of alcohol consumption and the prediction of the probability of an alcohol use disorder. The reason to merge former-smokers and non-smokers (vs. active smokers) was a clinical decision, since many risks of smoking such as cancer risks are significantly reduced after quitting smoking, especially at an early age (US Centers for Disease Control and Prevention (CDC). 2020; Rigotti et al., 2022). *Physical activity* was assessed with questions of the validated German “Fragebogen zur Erfassung des Gesundheitsverhaltens (FEG)” (questionnaire for recording health behavior) (Dlugosch & Krieger, 1995). In the original version, the questionnaire consists of eight areas: diet, physical activity, alcohol, smoking, medication, sleep, general well-being/problems and coping with health and illness. Due to the modular structure of the FEG, the individual areas can also be assessed separately.

### Clinical factors and family history

*Lower urinary tract symptoms (LUTS)* were categorized into no to mild symptoms (International Prostate Symptom Score (IPSS) ≤ 7) vs. moderate to severe symptoms (IPSS > 7), which is a validated and clinically used cut-off score in urology practice to detect symptomatic men (assessed with the validated German version (Badía et al., 1997) of the International Prostate Symptom Score (IPSS) (Barry et al., 2017). LUTS can be classified into two main categories: storage and voiding symptoms. Storage symptoms include frequency, urgency, urge incontinence, and nocturia. Voiding symptoms encompass straining, a poor and/or intermittent stream, prolonged micturition, a sensation of incomplete bladder emptying, and dribbling (Lepor, 2005). Self-reported *previous PSA tests* and *previous digital rectal examinations* were assessed in the clinical interview. *Family history* included diagnosed cancers of the participants themselves (*urological* and *non-urologic cancers*), as well as diagnosed cancers of their family members (*PCa* and *other cancers*).

### Psychological factors

*Health-related quality of life* was assessed using the Physical Health Composite Score and the Mental Health Composite Score of the Short Form Health Survey (SF-12) and categorized into higher and lower *physical/mental health status* (Ware et al., 1996, 1998). Due to missing validated cut-offs in the literature, we defined the cut-off as mean minus one standard deviation. Symptoms of *depression* and *anxiety* were assessed by the validated German version of the two-question screening tools Patient Health Questionnaire-2 and Generalized Anxiety Disorder-2, which are rated on a 4-point Likert scale. A summary score ≥ 3 indicates clinical levels of depression and anxiety, respectively (Löwe et al., 2010). Further psychological factors included *perceived ambiguity about PCa prevention recommendations* (“There are so many different recommendations about preventing prostate cancer, it is hard to know which one to follow.”) (Han et al., 2007), *perceived preventability of PCa* (“There is not much you can do to lower your chances of getting prostate cancer.”) (Han et al., 2007), and *perceived severity about developing PCa* (“Developing prostate cancer would be one of the worst things that could happen to me.”) (Vadaparampil et al., 2004). All 3 items were rated on a 4-point Likert scale and categorized in disagree (1–2) and agree (3–4), according to the original studies.

### Worry about PCa and risk perception

*Worry about PCa* was assessed with the following question: “During the past month, how worried or concerned have you been about having or getting prostate cancer?” (Wallner et al., 2008). Answers were rated on a 5-point Likert scale, dichotomized and categorized as “sometimes or (very) often” (3–5) vs. “not at all or rarely” (1–2) following previous study procedures (Wallner et al., 2008).

Risk perception was assessed by questions taken from Shavers et al. and rated on a 5-point Likert scale (Shavers et al., 2009). *Absolute risk perception* was assessed with the following question: “How likely do you think it is that you will develop prostate cancer in the future?“. Answers were dichotomized and categorized as “somewhat to very high” (4–5) vs. “very low to moderate” (1–3) following previous study procedures (Shavers et al., 2009). *Comparative risk perception* was assessed with the following question: “Compared to the average man of your age, how high is your risk of developing prostate cancer?“. Answers were dichotomized and categorized as “somewhat to much higher risk” (4–5) vs. “much lower to equal” (1–3) following previous study procedures (Shavers et al., 2009). Overall, since our study approach was exploratory in nature and the above-mentioned literature supports and favors dichotomization of these variables, we decided to follow previous study procedures and dichotomized the outcome variables (i.e., absolute/comparative risk perception and worry about PCa to allow a comparison of our results with those from previous studies. Further, the implementation of an ordinal logistic regression analysis with proportional odds was tested. However, this showed that the proportional odds assumption was not fulfilled in the models calculated for the three outcome variables.

### Statistical analysis

Descriptive statistics calculating counts and percentages for categorical variables were used to present participants’ characteristics. To identify and assess factors associated with worry about PCa, absolute risk perception, and comparative risk perception multivariable logistic regression models with backward elimination (elimination level of 5%) and ORs (odds ratios) with 95% CI (confidence intervals) were calculated. Variables used for the regression analyses are depicted in Table 3. All tests were two-sided, and data analyses were conducted using the Statistical Analysis System (SAS), version 9.4 (SAS Institute Inc., Cary, NC, USA).

## Results

### Characteristics of the study sample

Table 1 depicts the sociodemographic, lifestyle, clinical, and psychological characteristics as well as the family history of the study population. When surveyed, 17.2% reported having had a previous PSA test and 18.8% had a positive family history of PCa. Prevalence of moderate and severe LUTS (IPSS > 7) was 11.0%. About one third of the men perceived high ambiguity about PCa prevention recommendations (31.9%) and low preventability of PCa (33.1%) (Table 1).

**Table 1:** Baseline characteristics of the study sample of 45-year-old men

	*n*	%
Total	33,476	100.0
Sociodemographic factors		
Level of education		
Low	4,047	12.4
Intermediate	8,112	24.9
High	20,401	62.7
Partnership		
Yes	28,118	86.8
No	4,286	13.2
Health insurance		
Private	9,096	27.3
Statutory	24,223	72.7
Subjective economic situation		
Good	25,416	76.3
Poor	7,893	23.7
Lifestyle factors		
Body Mass Index (kg/m²)		
< 30.0	26,748	80.2
≥ 30.0	6,594	19.8
Waist circumference (cm)		
≤ 102	24,041	73.4
> 102	8696	26.6
Alcohol consumption		
High (≥ 5 alcoholic beverages on ≥ 3 days/week)	1,327	4.2
Low to moderate	23,738	74.3
No	6,876	21.5
Active smoking		
Current smoker	6,068	18.6
Former smoker	9,582	29.4
Non-smoker	16,942	52.0
Physical activity		
Yes (minimum of 30 min ≥2 times/week)	26,364	80.7
No	6,318	19.3
Clinical factors and family history		
Lower urinary tract symptoms		
No, mild symptoms (IPSS ≤ 7)	28,439	89.0
Moderate, severe symptoms (IPSS > 7)	3,511	11.0
Previous PSA test before study enrollment		
Yes	5,493	17.2
No	26,519	82.8
Previous digital rectal examination before study enrollment		
Yes	12,097	38.0
No	19,759	62.0
History of urologic cancer		
Yes	275	0.8
No	32,792	99.2
History of non-urologic cancer		
Yes	838	2.5
No	32,229	97.5
Family history of PCa		
Yes	6,112	18.8
No	26,462	81.2
Family history of other cancers		
Yes	18,769	59.0
No	13,020	41.0
Psychological factors		
Health-related quality of life		
Physical health status (PCS)		
High	26,593	89.2
Low	3,231	10.8
Mental health status (MCS)		
High	25,978	87.1
Low	3,846	12.9
Depression (PHQ-2)		
Yes (≥ 3)	1,750	5.3
No (< 3)	31,367	94.7
Anxiety (GAD-2)		
Yes (≥ 3)	1,763	5.3
No (< 3)	31,305	94.7
Perceived ambiguity		
High	10,200	31.9
Low	21,739	68.1
Perceived preventability		
High	22,035	66.9
Low	10,926	33.1
Perceived severity		
High	16,105	48.8
Low	16,908	51.2

IPSS = International Prostate Symptom Score; PSA = prostate-specific antigen; PCS = physical component summary; MCS = mental component summary; PHQ-2 = Patient Health Questionnaire-2; GAD-2 = Generalized Anxiety Disorder-2

7.3% had sometimes or (very) often worries about PCa, 3.7% perceived their absolute risk of developing PCa in the future as somewhat high/very high, and 9.9% perceived their comparative risk as somewhat higher/much higher compared to the average men of the same age (Table 2).

**Table 2:** Frequency distribution of worry about prostate cancer, absolute risk perception, and comparative risk perception

	*n*	%
**Total**	33,476	100.0
Worry about prostate cancer		
Not at all	24,493	73.2
Rarely	6,528	19.5
Sometimes	2,155	6.4
Often	261	0.8
Very often	39	0.1
Absolute risk perception		
Very low	6,927	20.7
Somewhat low	15,067	45.0
Moderate	10,237	30.6
Somewhat high	1,126	3.4
Very high	119	0.3
Comparative risk perception		
Much lower	5,069	15.1
Somewhat lower	8,502	25.4
Equal	16,608	49.6
Somewhat higher	3,004	9.0
Much higher	293	0.9

### Multivariable logistic regression analysis

Significant factors with the highest odds of **worry about PCa** included the clinical factors LUTS (OR 3.00, 95% CI 2.63–3.42) and a positive family history of PCa (OR 2.35, 95% CI 2.08–2.65) as well as the psychological factor high perceived severity about developing PCa (OR 2.01, 95% CI 1.80–2.26). Further factors associated were high perceived ambiguity about PCa prevention recommendations (OR 1.33, 95% CI 1.19–1.48) and a previous PSA test (OR 1.21, 95% CI 1.05–1.38). Important preventive factors included a better mental health status (OR 0.63, 95% CI 0.55–0.73) and sociodemographic factors such as a higher level of education (OR 0.74, 95% CI 0.62–0.88; OR 0.62, 95% CI 0.53–0.73), and a private health insurance status (OR 0.76, 95% CI 0.67–0.88) which were associated with less worry about PCa.

The factors with the highest odds of **absolute risk perception** were a positive family history of PCa (OR 15.13, 95% CI 12.73–17.97) and clinical factors LUTS (OR 2.09, 95% CI 1.71–2.56) and a previous PSA test (OR 1.82, 95% CI 1.55–2.14). Further factors associated with absolute risk perceptions included anxiety (OR 1.64, 95% CI 1.21–2.22), higher waist circumference (OR 1.34, 95% CI 1.13–1.59), a positive family history of other cancers (OR 1.39, 95% CI 1.19–1.63), and high perceived preventability of PCa (OR 1.56, 95%CI 1.34–1.81). Better mental health status was associated with less absolute risk perception (OR 0.67, 95% CI 0.53–0.83).

Important factors with the highest odds of **comparative risk perception** included aspects of family history and clinical factors such as a positive family history of PCa (OR 9.69, 95% CI 8.76–10.72), a positive history of urological cancers (OR 3.85, 95% CI 2.63–5.63), LUTS (OR 2.41, 95% CI 2.10–2.76), a positive history of non-urologic cancers (OR 1.97, 95% CI 1.52–2.54), a previous PSA test (OR 1.66, 95% CI 1.48–1.88), a positive family history of other cancers (OR 1.54, 95% CI 1.39–1.71) as well as a high level of education (OR 1.50, 95% CI 1.25–1.80). Further factors associated with comparative risk perception included all lifestyle factors such as a higher BMI (OR 1.31, 95% CI 1.11–1.54), higher waist circumference (OR 1.26, 95% CI 1.00-1.59), high alcohol consumption (OR 1.26, 95% CI 1.00-1.59), active smoking (OR 1.28, 95% CI 1.13–1.45) and clinical and psychological factors (previous digital rectal examination (OR 1.23, 95% CI 1.11–1.37), depression (OR 1.27, 95% CI 1.00-1.60), and anxiety (OR 1.37, 95% CI 1.09–1.72)). Preventive factors included both a better physical (OR 0.75, 95% CI 0.64–0.87) and mental (OR 0.79, 95% CI 0.67–0.92) health status as well as physical activity (OR 0.83, 95% CI 0.73–0.93), which were associated with less comparative risk perception (Table 3).

**Table 3:** Multivariable logistic regression analysis with backward elimination of worry about PCa, absolute risk perception, and comparative risk perception

	Worry about PCa^a^			Absolute risk perception^b^			Comparative risk perception^c^		
	OR	95%CI	p value	OR	95%CI	p value	OR	95%CI	p value
Sociodemographic factors									
Level of education (intermediate vs. low)	0.74	0.62–0.88	< 0.001	0.82	0.60–1.13	0.230	0.98	0.80–1.20	0.817
Level of education (high vs. low)	0.62	0.53–0.73	< 0.001	1.13	0.86–1.49	0.396	1.50	1.25–1.80	< 0.001
Partnership (yes vs. no)	-	-	-	-	-	-	-	-	-
Health insurance (private vs. statutory)	0.76	0.67–0.88	< 0.001	-	-	-	-	-	-
Subjective economic situation (poor vs. good)	-	-	-	-	-	-	-	-	-
Lifestyle factors									
Body Mass Index (kg/m²) (≥ 30.0 vs. <30.0)	-	-	-	-	-	-	1.31	1.11–1.54	0.001
Waist circumference (cm) (> 102 vs. ≤102)	1.13	1.01–1.28	0.040	1.34	1.13–1.59	< 0.001	1.26	1.09–1.46	0.002
Alcohol consumption (high vs. moderate, no)	-	-	-	-	-	-	1.26	1.00-1.59	0.047
Active smoking (yes vs. no)	1.17	1.02–1.33	0.025	-	-	-	1.28	1.13–1.45	< 0.001
Physical activity (yes vs. no)	-	-	-	-	-	-	0.83	0.73–0.93	0.002
Clinical factors and family history									
Lower urinary tract symptoms (IPSS > 7 vs. IPSS ≤ 7)	3.00	2.63–3.42	< 0.001	2.09	1.71–2.56	< 0.001	2.41	2.10–2.76	< 0.001
Previous PSA test (yes vs. no)	1.21	1.05–1.38	0.007	1.82	1.55–2.14	< 0.001	1.66	1.47–1.87	< 0.001
Previous digital rectal examination (yes vs. no)	-	-	-	-	-	-	1.23	1.11–1.37	< 0.001
History of urologic cancer (yes vs. no)	-	-	-	-	-	-	3.85	2.63–5.63	< 0.001
History of non-urologic cancer (yes vs. no)	-	-	-	-	-	-	1.97	1.52–2.54	< 0.001
Family history of PCa (yes vs. no)	2.35	2.08–2.65	< 0.001	15.13	12.73–17.97	< 0.001	9.69	8.76–10.72	< 0.001
Family history of other cancers (yes vs. no)	-	-	-	1.39	1.19–1.63	< 0.001	1.54	1.39–1.71	< 0.001
Psychological factors									
Health-related quality of life									
Physical health status (PCS) (high vs. low)	-	-	-	-	-	-	0.75	0.64–0.87	< 0.001
Mental health status (MCS) (high vs. low)	0.63	0.55–0.73	< 0.001	0.67	0.53–0.83	< 0.001	0.79	0.67–0.92	0.003
Depression (PHQ-2) (yes (≥ 3) vs. no (< 3))	-	-	-	-	-	-	1.27	1.00-1.60	0.048
Anxiety (GAD-2) (yes (≥ 3) vs. no (< 3))	-	-	-	1.64	1.21–2.22	0.001	1.37	1.09–1.72	0.007
Perceived ambiguity (high vs. low)	1.33	1.19–1.48	< 0.001	-	-	-	-	-	-
Perceived preventability (low vs. high)	-	-	-	1.56	1.34–1.81	< 0.001	-	-	-
Perceived severity (high vs. low)	2.01	1.80–2.26	< 0.001	-	-	-	-	-	-

CI = confidence interval; IPSS = International Prostate Symptom Score; OR = odds ratio; PCa = prostate cancer; PSA = prostate-specific antigen; PCS = physical component summary; MCS = mental component summary; PHQ-2 = Patient Health Questionnaire-2; GAD = Generalized Anxiety Disorder-2

^a^Worry about PCa was rated on a 5-point Likert scale and dichotomized in “sometimes or (very) often” (3–5) vs. “not at all or rarely” (1–2)

^b^Absolute risk perception was rated on a 5-point Likert scale and dichotomized in “somewhat to very high” (4–5) vs. “very low to moderate” (1–3)

^c^Comparative risk perception was rated on a 5-point Likert scale and dichotomized in “somewhat to much higher risk” (4–5) vs. “much lower to equal” (1–3)

## Discussion

The aim of the current study was to assess prevalence and factors associated with worry about PCa and risk perception in a large, community-based sample of middle-aged men Multivariable logistic regression analyses revealed that important factors of worry about PCa were LUTS, a positive family history of PCa, and high perceived severity about developing PCa. Important factors of absolute risk perception were also a positive family history of PCa and LUTS, and additionally previous PSA testing. Important factors of comparative risk perception were again a positive family history of PCa and LUTS, but also a positive history of own urological cancers and previous PSA testing.

In general, worry about PCa and risk perception were lower among the middle-aged men of this study compared to available research in population-based studies (Shavers et al., 2009; Wallner et al., 2008). As such, prevalence of worry about PCa was slightly lower in this study compared to a randomly selected cohort of American men of the Olmsted County Study (7.3% vs. 10.3%) (Wallner et al., 2008). However, the American sample had a median age of 51.9 years, which might be one reason for the higher prevalence, since the risk of PCa increases with higher age. Only 3.7% of men surveyed in the PROBASE trial perceived their future risk of developing PCa as somewhat or very high compared to 14.5% in a nationally representative sample of American men of the Health Information National Trends Survey (Shavers et al., 2009). Regarding comparative risk perception, 9.9% of men surveyed in the PROBASE trial perceived their comparative risk as somewhat or much higher as the average men of their age. Similarly, the Health Information National Trends Survey reported higher numbers of comparative risk perception (12.9%) (Shavers et al., 2009). While both aforementioned studies, i.e., the Olmsted County Study and the Health Information National Trends Survey (Shavers et al., 2009; Wallner et al., 2008), utilized large population-based cohorts either through a telephone or an in-home interview, the sample of the PROBASE trial consisted of men taking part in a randomized PCa screening trial. There is ample evidence that risk perception and health behavior are strongly associated (Beebe-Dimmer et al., 2004; Brewer et al., 2004; McDowell et al., 2009; Vadaparampil et al., 2004). According to the Risk Reappraisal Hypothesis (Brewer et al., 2004) performance of a health protective behavior, i.e. PCa screening, results in a lowering of risk perceptions. Another explanation for lower risk perceptions among participants of a PCa screening trial relates to research on insurance and risk perceptions. In a comparative study, men who were insured (e.g., health, travel, car) felt they were less likely to suffer problems in the future compared to their counterparts (Tykocinski, 2008). Taken together, taking part in a PCa screening trial might equally lead to lower risk perceptions and cancer worry resulting in a healthy screenee bias.

The main results of the current study showed that a positive family history of PCa was an important factor of worry about PCa and was associated with a 15- and 10-fold increase in the odds of absolute and comparative risk perception, respectively. These results are in line with previous research (Bratt et al., 2000; McDowell et al., 2013; Miller et al., 2001; Sweetman et al., 2006). Interestingly, a positive family history of PCa as well as of other cancers showed significant results for worry about PCa and absolute risk perception compared to a personal history of either urologic cancers or non-urologic cancers, which is consistent with results found in previous studies in colon cancer research (Hay et al., 2006). Witnessing a close relative going through treatment for cancer might lead to feeling vulnerable through a process of vicarious learning (Schwarzer, 1994). Another explanation is that people may believe they are genetically predisposed if a close relative has had PCa. Since a positive family history of PCa, in addition to age and African ancestry, is an objective risk factor for the disease, these results are hardly surprising. Rather, the absolute number of men experiencing worry about PCa (7.3%) or perceiving their absolute risk as somewhat high to very high (3.7%) or perceiving their comparative risk as somewhat higher to much higher (9.9%) seems low compared to the number of men with a positive family history (18.8%) in this sample. Such inconsistencies between the actual and perceived risk have been likewise reported relating to African Americans (Shavers et al., 2009) as well as older men with a PCa family history (McDowell et al., 2013) who are both often not aware of their increased risk of developing PCa. Therefore, there is a crucial need in risk communication to address this underestimation of risk.

LUTS was an important factor in association with worry about PCa and both absolute and comparative risk perception. Although several studies have shown that men with LUTS are at no greater risk of PCa than asymptomatic men of the same age (Young et al., 2000), many men express fears about PCa in relation to their LUTS (Brown et al., 2003). Interestingly, in a population-based sample of US American men, bowel symptoms were also strongly associated with higher perceived risk of colorectal cancer (Robb et al., 2004). These findings are of some concern, given the fact that PCa as well as colorectal cancer are often asymptomatic until an advanced stage and early detection plays an important role in the curative treatment of these cancers. These misperceptions surrounding the effect of LUTS on perceived risk need to be addressed in risk communication.

Previous PSA testing before the study enrollment at the age of 45 was associated with both higher absolute and comparative risk perception, whereas previous digital rectal examination showed no association. Both results are in line with previous results in African American men (Bloom et al., 2006). Apparently, the higher risk perception of developing PCa is a significant reason that affected men undergo PSA testing, even before the age of 45, which is not recommended for men without elevated risk by any guideline. Interestingly, an abnormal fear of cancer seems not to be the reason, since worry about PCa was not associated with previous PSA testing.

Of the psychological factors, high perceived severity of developing PCa was associated with a 2-fold increase in the odds of being worried about PCa. To our knowledge, this is the first time this factor is being investigated as a predictor of worry about PCa. However, it seems plausible that men fear a potential disease which is described by themselves as one of the worst things that could happen. However, risk perceptions were not associated with high perceived severity showing that these men do not have inadequately high levels of risk perception, since they are not at increased risk in general. The fact that perceived severity was associated with worry but not with risk perceptions is supported by previous findings in the literature. For instance, in participants receiving information about a genetic test of lifetime risks for various diseases (e.g., diabetes, heart disease, colon cancer) worry was correlated with perceived severity and likelihood of these diseases (Cameron et al., 2009). Analysis of further psychological factors revealed that higher perceived ambiguity about PCa prevention recommendations was associated with higher worry about PCa, but showed no association with absolute and comparative risk perceptions. Ambiguity, defined as uncertainty about the reliability and credibility of information about risks and potential decision outcomes, has been shown to trigger specific psychological and behavioral effects. Notably, it prompts people to view risks and possible outcomes more pessimistically and to steer away from decision making (Ellsberg, 1961). For instance, perceived ambiguity about other cancer types such as lung, colon, and skin cancer is inversely associated with perceptions of the preventability and with cancer-specific risk-modifying behaviors including cancer screening (e.g., smoking abstinence, sigmoidoscopy–colonoscopy testing, sunscreen use). However, for lung cancer only, perceived ambiguity has been shown to be associated with absolute risk perception and cancer worry. Colon and skin cancer showed no relationship between perceived ambiguity and risk perceptions and cancer worry, respectively (Han et al., 2007). These cancer-specific differences underline the importance of understanding how ambiguity perceptions may influence people’s behaviors and cognitions such as perceptions regarding the risk and controllability of a disease, and the risks of both choosing or avoiding a disease-protective intervention (Han et al., 2007; Leventhal et al., 2016). In PCa, it seems that the large number of existing prevention recommendations lead to increased cancer worry, while risk perceptions may not influenced. However, higher perceived preventability was associated with higher absolute risk perceptions, but not with cancer worry. This is in line with previous results in first-degree-relatives of colorectal cancer patients showing that a higher perceived risk was associated with believing that colorectal cancer cannot be prevented. Since most of them overestimated their actual cancer risk, perceived risk changed after counselling (Codori et al., 2005). In this context, adequate counselling and risk communication with patients is crucial to avoid misperceptions regarding the true risk.

From the sociodemographic variables, a higher level of education was associated with less worry about PCa. This result seems somewhat plausible since the prevalence of PCa in 45-year-old men is in general low and educated people might be aware of this. Further, previous research supports this inverse association in more than 2,000 men and women ≥ 50 years old with no history of cancers regarding breast, prostate, and colon cancer worry (McQueen et al., 2008). In the current study, absolute risk perception was not related to level of education, which is in line with other population-based studies (McQueen et al., 2008; Shavers et al., 2009). However, the fact that higher comparative risk perception was associated with a high level of education among participants of the current study remains unclear. Compared to the literature, divergent results can be found. Conversely, one study found that men with a low level of education had even higher comparative risk perceptions (Shavers et al., 2009). Previous research on comparative risk perception showed that it is more likely to be associated with worry and knowledge than absolute risk perception (Dillard et al., 2011; McCaul et al., 2003). However, since different assessments of the factor educational level were used, comparisons are difficult and valid conclusions can hardly be drawn. The other sociodemographic factors such as partnership and subjective economic situation showed likewise to the literature no significant results (Beebe-Dimmer et al., 2004; McQueen et al., 2008; Shavers et al., 2009).

The assessment of lifestyle factors showed that all risk factors (i.e., higher body mass index, higher waist circumference, high alcohol consumption, active smoking, and no physical activity) were associated with higher comparative risk perception. Interestingly, only higher waist circumference was associated with additionally both a higher absolute risk perception and worry about PCa, and active smoking was only associated with worry about PCa. This fact is somewhat disturbing, since all of these risk factors are related to a higher risk of PCa (Freedland & Aronson, 2004; Huncharek et al., 2010; Macke & Petrosyan, 2022; Shephard, 2017). Obviously, affected men are aware of their increased comparative risk, however, they do not perceive a higher absolute risk and also no or little worry about PCa, which highlights the need for more awareness in preventive measures.

To date, the current study is the largest community-based investigation on a broad set of sociodemographic, lifestyle, psychological, and clinical factors as well as family history in relation to worry about PCa and risk perceptions. Comparable data are only available for other cancers such as colon, breast or lung cancer (Hay et al., 2006; Lebrett et al., 2022; Zajac et al., 2006). The large, community-based sample provides a good generalizability and important insights into the predictors of health behavior of middle-aged men at the beginning of PCa screening. However, there are limitations to this study that need to be considered in interpreting the results. First, due to missing information in further variables assessed, 12,249 out of 33,476 men were not included in the regression analyses. However, the dropout analysis revealed only marginal differences (all < 8%) between the two groups. Notably, all lifestyle, clinical, and especially psychological factors as well as family history showed no to only marginal differences (< 3.5%) between the two groups. Second, the cross-sectional design does not allow to draw causal conclusions. Third, since the PROBASE trial is a PCa screening trial health-concerned men might be overrepresented, as well as men who might be suffering from prostate symptoms resulting in a recruitment bias. Further, since recruitment criteria of the PROBASE trial included 45-year-old men results of the current study are limited to middle-aged men and cannot be generalized to men of other ages or women; however, this rigorous participant selection allows excellent comparability and precise factor evaluation. Fourth, due to the low number of study participants with an even lower response rate, we had to exclude men with African and Asian ancestry, which is a notable limitation, since men with African ancestry are at increased risk of PCa. Therefore, our results are limited to White men.

## Conclusion

This study is the first to explore important factors associated with worry about PCa and risk perceptions in a large community-based sample of middle-aged men using a multivariable approach to investigate a broad range of demographic, lifestyle, clinical, and psychological factors as well as family history. A positive family history of PCa, which is a well-known risk factor for PCa, strongly influenced risk perception. Since the absolute number of men affected by a positive family history was in total much higher than the absolute number of men with worry about PCa or higher risk perceptions, there is a crucial need to improve risk communication, addressing this underestimation of risk. Furthermore, although PCa is often asymptomatic until an advanced stage and men with LUTS are at no greater risk of PCa than asymptomatic men of the same age, LUTS was likewise an important factor associated with worry about PCa and risk perception. These misperceptions need to be addressed in risk communication with patients.

## Electronic supplementary material

Below is the link to the electronic supplementary material.

Supplementary Material 1

## Data Availability

All original data are available upon reasonable personal request to r.kaaks@dkfz.de.
