# Supplementary material for: Worry about prostate cancer and risk perception among middle-aged men: results from the PROBASE trial
Source: J Behav Med. 2025 Mar 5;48(3):464–77. doi: 10.1007/s10865-025-00559-w (PMC12078363; doi:10.1007/s10865-025-00559-w)
Supplement: Supplementary file 1 — Supplementary Material 1 [file 10865_2025_559_MOESM1_ESM.docx]

| **Supplementary Table 1.** Dropout analysis of men included in the regression analyses (n = 21,227) and those who were excluded (n = 12,249) due to missing information | | | | |
| --- | --- | --- | --- | --- |
|  | Included | | Excluded | |
|  | n | % | n | % |
| Total | 21,227 | 100.0 | 12,249 | 100.0 |
| Sociodemographic factors |  |  |  |  |
| Level of education |  |  |  |  |
| Low | 2,232 | 10.5 | 1,815 | 16.0 |
| Intermediate | 5,136 | 24.2 | 2,976 | 26.3 |
| High | 13,859 | 65.3 | 6,542 | 57.7 |
| Missing |  |  | 916 |  |
| Partnership |  |  |  |  |
| Yes | 18,642 | 87.8 | 9,476 | 84.8 |
| No | 2,585 | 12.2 | 1,701 | 15.2 |
| Missing |  |  | 1,072 |  |
| Health insurance |  |  |  |  |
| Private | 6,138 | 28.9 | 2,958 | 24.5 |
| Statutory | 15,089 | 71.1 | 9,134 | 75.5 |
| Missing |  |  | 157 |  |
| Subjective economic situation |  |  |  |  |
| Good | 16,707 | 78.7 | 8,709 | 72.1 |
| Poor | 4,520 | 21.3 | 3,373 | 27.9 |
| Missing |  |  | 167 |  |
| Lifestyle factors |  |  |  |  |
| Body Mass Index (kg/m²) |  |  |  |  |
| <30.0 | 17,110 | 80.6 | 9,638 | 79.5 |
| ≥30.0 | 4,117 | 19.4 | 2,477 | 20.5 |
| Missing |  |  | 134 |  |
| Waist circumference (cm) |  |  |  |  |
| ≤102 | 15,636 | 73.7 | 6,223 | 73.0 |
| >102 | 5,591 | 26.3 | 3,105 | 27.0 |
| Missing |  |  | 739 |  |
| Alcohol consumption |  |  |  |  |
| High | 855 | 4.0 | 472 | 4.4 |
| Low to moderate/no | 20,372 | 96.0 | 10,242 | 95.6 |
| Missing |  |  | 1,535 |  |
| Active smoking |  |  |  |  |
| Yes (current smoker) | 3,789 | 17.9 | 2,279 | 20.1 |
| No (former smoker/non-smoker) | 17,438 | 82.1 | 9,086 | 79.9 |
| Missing |  |  | 884 |  |
| Physical activity |  |  |  |  |
| Yes (minimum of 30 minutes ≥2 times/week) | 17,142 | 80.8 | 9,222 | 80.5 |
| No | 4,085 | 19.2 | 2,233 | 19.5 |
| Missing |  |  | 794 |  |
| Clinical factors and family history | | | | |
| Lower urinary tract symptoms |  |  |  |  |
| No, mild (IPSS ≤7) | 19,027 | 89.6 | 9,412 | 87.8 |
| Moderate, severe (IPSS >7) | 2,200 | 10.4 | 1,311 | 12.2 |
| Missing |  |  | 1,526 |  |
| Previous PSA test |  |  |  |  |
| Yes | 3,805 | 17.9 | 1,688 | 15.7 |
| No | 17,422 | 82.1 | 9,097 | 84.3 |
| Missing |  |  | 1,464 |  |
| Previous digital rectal examination |  |  |  |  |
| Yes | 8,109 | 38.2 | 3,988 | 37.5 |
| No | 13,118 | 61.8 | 6,641 | 62.5 |
| Missing |  |  | 1,620 |  |
| History of urologic cancer |  |  |  |  |
| Yes | 176 | 0.8 | 99 | 0.8 |
| No | 21,051 | 99.2 | 11,741 | 99.2 |
| Missing |  |  | 409 |  |
| History of non-urologic cancer |  |  |  |  |
| Yes | 552 | 2.6 | 286 | 2.4 |
| No | 20,675 | 97.4 | 11,554 | 97.6 |
| Missing |  |  | 409 |  |
| Family history of PCa |  |  |  |  |
| Yes | 3,999 | 18.8 | 2,113 | 18.6 |
| No | 17,228 | 81.2 | 9,234 | 81.4 |
| Missing |  |  | 902 |  |
| Family history of other cancers |  |  |  |  |
| Yes | 12,629 | 59.5 | 6,140 | 58.1 |
| No | 8,598 | 40.5 | 4,422 | 41.9 |
| Missing |  |  | 1,687 |  |
| Psychological factors |  |  |  |  |
| Health-related quality of life |  |  |  |  |
| Physical health status (PCS) |  |  |  |  |
| High | 19,052 | 89.8 | 7,541 | 87.7 |
| Low | 2,175 | 10.2 | 1,056 | 12.3 |
| Missing |  |  | 3,652 |  |
| Mental health status (MCS) |  |  |  |  |
| High | 18,566 | 87.5 | 7,412 | 86.2 |
| Low | 2,661 | 12.5 | 1,185 | 13.8 |
| Missing |  |  | 3,652 |  |
| Depression (PHQ-2) |  |  |  |  |
| Yes (≥3) | 1,006 | 4.7 | 744 | 6.3 |
| No (<3) | 20,221 | 95.3 | 11,146 | 93.7 |
| Missing |  |  | 359 |  |
| Anxiety (GAD-2) |  |  |  |  |
| Yes (≥3) | 1,051 | 5.0 | 712 | 6.0 |
| No (<3) | 20,176 | 95.0 | 11,129 | 94.0 |
| Missing |  |  | 408 |  |
| Perceived ambiguity |  |  |  |  |
| High | 6,562 | 30.9 | 3,638 | 34.0 |
| Low | 14,665 | 69.1 | 7,074 | 66.0 |
| Missing |  |  | 1,537 |  |
| Perceived preventability |  |  |  |  |
| High | 14,206 | 66.9 | 7,829 | 66.7 |
| Low | 7,021 | 33.1 | 3,905 | 33.3 |
| Missing |  |  | 515 |  |
| Perceived severity |  |  |  |  |
| High | 10,196 | 48.0 | 5,909 | 50.1 |
| Low | 11,031 | 52.0 | 5,877 | 49.9 |
| Missing |  |  | 463 |  |
| IPSS = International Prostate Symptom Score; PSA = prostate-specific antigen; PCS = physical component summary; MCS = mental component summary; PHQ-2 = Patient Health Questionnaire-2; GAD-2 = Generalized Anxiety Disorder-2 | | | | |
